# Why should pediatric rheumatology be recognized as a separate subspecialty: an open letter to medical councils and government agencies

**DOI:** 10.1186/1546-0096-5-21

**Published:** 2007-11-21

**Authors:** Charles H Spencer

**Affiliations:** 1 Department of Pediatrics, Nationwide Children's Hospital/Ohio State University, Columbus, Ohio USA

## Abstract

Some pediatric rheumatologists in the West may take for granted that pediatric rheumatology (PR) is a recognized subspecialty. Yet pediatric rheumatology has been accepted as a subspecialty in the United States only since 1990. There are still countries where many pediatric subspecialties are not given official recognition and support, including PR. This lack of recognition delays and impedes the development of PR, appropriate musculoskeletal and rheumatic teaching in medical schools, and optimal diagnosis and treatment for children with these illnesses. In the opinion of editorial staff, each country where pediatric rheumatology is reasonably well developed as a subspecialty has an obligation to help our pediatric rheumatologists elsewhere gain recognition, support, and respect. The Pediatric Rheumatology European Society (PReS) and the Pediatric Rheumatology International Trial Organization (PRINTO) have been leaders in this effort, but in many countries, pediatric rheumatology is still not recognized. This editorial offers rationales and justifications for medical and governmental entities accrediting pediatric rheumatology as a separate subspecialty that may aid in these efforts.

## Editorial

I am writing to you in support of an application of new pediatric rheumatologists in your country to have their subspecialty accredited by your governmental agency or medical council and to receive governmental and university support. I suggest to you that their application should be approved in your country for the sake of children with rheumatic disease.

I have five reasons for supporting their application for recognition as a pediatric subspecialty distinct from pediatrics and adult rheumatology:

1) Contrary to common belief, there are substantial numbers of children with rheumatic disease in your country and around the world. The best prevalence figures are that 1–4/1000 children and adolescents have chronic arthritis due to juvenile idiopathic arthritis. [1] For example, in a country of 10 million, there are approximately 3 million children; that translates into 5000 to 10,000 children with chronic arthritis diseases including juvenile idiopathic arthritis, lupus, dermatomyositis, sarcoidosis, and others. Where are they? They are there but they are often invisible, flying beneath the radar. They are often misdiagnosed and/or slow to be diagnosed and treated. These diseases do not often kill but they can cause much suffering and disability, especially when not properly diagnosed and treated. The cost to your society and gross national product can be considerable. Yes, there are many competing medical costs for children with malnutrition, diarrhea illnesses, HIV, tuberculosis, severe poverty, and other health challenges. But children with arthritis illnesses also deserve your help.

2) There are an inadequate number of pediatric rheumatologists in your country and most countries for patient care. By a recent guideline developed in the United Kingdom, a country such as yours needs one pediatric rheumatologist per 300,000–500,000 population of children just for clinical care. At a current population of 10 million, from all available evidence, your country needs a minimum of 6 pediatric rheumatologists to perform the necessary clinical duties in the medical schools and medical centers and would need an additional 6 to provide an acceptable level of teaching and research in your medical colleges, with at least one per medical college. To get to this level of 12 pediatric rheumatologists or more in a country of 10 million, we believe that at a minimum the pediatric rheumatology subspecialty must be recognized. It is optimal if a national center for pediatric rheumatology be established with a training program to develop new pediatric rheumatologists. It is our experience that when pediatric rheumatology centers are not established, these children are not properly diagnosed and treated.

3) Education about the medical musculoskeletal problems in children suffers without sufficient pediatric rheumatologists available to teach medical students and residents and update pediatricians. The musculoskeletal exam of children is underemphasized in medical schools and resident training programs worldwide. In the UK a study that reviewed the documentation of 257 admission pediatric history and physicals noted that only 4% had a musculoskeletal exam documented at all compared to >90% for the lung and heart. [2] Time and time again, we have noted that without a pediatric rheumatologist, no one provides an education on non-surgical musculoskeletal conditions (not even orthopedists). Young physicians may finish training with minimal exposure to these musculoskeletal and rheumatic diseases in kids and how to diagnose them, to the great disadvantage of children. It should be noted that 6% of pediatric practice outpatient visits may involve musculoskeletal pain problems [3] Primary care physicians who have not had training in a screening musculoskeletal exam such as pGALS are likely at a disadvantage. [4]

These concerns are not limited to pediatrics. Several studies have shown major deficiencies in the knowledge of musculoskeletal medicine and the musculoskeletal examination in the United States and there is no reason to believe that the musculoskeletal education is better elsewhere. [5,6] The American Association of Medical Colleges has urged medical colleges to develop a longitudinal educational program in musculoskeletal medicine through all years of medical school. [7] As noted by the World Health Organization in 2003, despite its low profile due to a minimal mortality rate, "musculoskeletal or rheumatic diseases are the major cause of morbidity throughout the world, having a substantial influence on health and quality of life, and inflicting an enormous burden on health systems." [8]

4) Adult rheumatologists or skilled general pediatricians are not the answer to fill this gap in education and patient care. Adult rheumatologists are internists and have limited training in caring for children and adolescents with rheumatic diseases; many have only one month of experience with children with musculoskeletal and rheumatic diseases in training, if that or are trying to learn by doing. They are often too busy caring for and teaching about adult rheumatic problems to focus on caring for kids and teaching about pediatric arthritis. They may consider pediatric rheumatic diseases to be very similar to the adult rheumatic diseases. Yet, in the view of many pediatric rheumatologists, pediatric rheumatic diseases and mimicking diseases are often quite different than those seen in adults (e.g., juvenile idiopathic arthritis, juvenile dermatomyositis, local scleroderma, Henoch-Schonlein purpura, periodic fever syndromes, juvenile sarcoidosis under age 10 years, Kawasaki disease, neonatal lupus, genetic skeletal diseases, metabolic diseases, malignancies) than diseases that are similar or identical (e.g., lupus, RF+ polyarticular JRA, systemic vasculitis, systemic sclerosis, sarcoidosis over 10 years of age).

There are also multiple pediatric issues that internists tend to have limited understanding of such as pain in children, use of anti-rheumatic drugs in children, the art of dealing with parents, school problems, transition into adulthood, and others. Unless the adult rheumatologist decides to increase his pediatric patients to 25% of his practice, he/she will not develop the expertise needed to provide standard of practice care and be able to maintain competency over a medical career.

True, there will never be prospective studies that validate that the quality of care given by pediatric rheumatologists is better. In my opinion, there is no need. It is self-evident in the growth of pediatric rheumatology and other pediatric subspecialties. In the end it comes down to who would you or other parents want to care for your child with arthritis, a pediatrician who does pediatric rheumatology more than 50% of the time, often 100% of the time, or an internist who sees only 5–10% children? Granted, there are areas of each country where the only rheumatologist available is an adult rheumatologist and their help is essential and appreciated-yet that is a stop-gap solution and development of pediatric rheumatology in every country is the only acceptable long term solution.

General pediatricians have much to know about many diseases. It is difficult for a primary care physician to become competent in diagnosing and managing these children with rheumatic disease unless he or she takes a 1–3 year pediatric rheumatology fellowship or learns the hard way by gradually seeing more and more patients until they represent >25% of the physician's practice. The primary care physician would need to seek continuing medical education at rheumatology meetings. This road has been followed by many pioneers in pediatric rheumatology and is being followed even now in some countries, but it is a difficult road, requiring time, effort, funding, and local support.

Finally, who educates the medical students and residents as well as practicing pediatricians and family physicians if there are no pediatric rheumatologists? Adult rheumatologists often have their own educational duties to teach internists about adult arthritis and do not dependably teach pediatricians and other child health care providers. Similarly, generalist pediatricians may often have higher priority educational areas to emphasize in their teaching programs and efforts.

5) The key to providing care for these children is developing the pediatric rheumatology (PR) subspecialty by recognition of PR as a legitimate subspecialty. This has already occurred in the US, Canada, and the UK and is happening now in Europe and Asia. But progress is too slow. In the US there are now 237 American Board of pediatric-certified pediatric rheumatologists and there has been a certification board exam available since 1992. Growth is not easy but it is speeding up. [9] For the benefit of the children, we need more pediatric rheumatologists in many other countries. We believe recognition of pediatric rheumatology is critical. A recent United States government study documented the immediate need for an increase of 30% pediatric rheumatology workforce in the US. The study provides excellent justification for support for this field in the US and in every country. [10] The peril of not providing a sufficient workforce of pediatric rheumatologists is eloquently described by a parent of a child with dermatomyositis in a recent article in the Washington Post newspaper. [11]

I admit to my bias and passion. I believe in pediatric rheumatology and its mission. We have all seen an enormous growth around the world of pediatric rheumatology with many countries recognizing pediatric rheumatology and aiding its development. But our work is only partially finished. For the benefit of children with arthritis diseases, I hope that you will officially recognize pediatric rheumatology in your country as a legitimate pediatric subspecialty and that you will support its development.

## Competing interests

Charles H. Spencer, MD is an Editor-in Chief of Pediatric Rheumatology with Alberto Martini, MD
